# Association analyses between urinary concentrations of multiple trace elements and gastric precancerous lesions and gastric cancer in Anhui province, eastern China

**DOI:** 10.3389/fpubh.2024.1423286

**Published:** 2024-08-15

**Authors:** Shiqing Qian, Fang Xu, Min Wang, Meng Zhang, Shaopeng Ding, Guoqing Jin, Xiaohui Zhang, Wenli Cheng, Li Wang, Yuting Zhu, Wuqi Wang, Princess Ofosuhemaa, Tingting Wang, Xiao Lin, Yu Zhu, Yaning Lv, Anla Hu, Wanshui Yang, Gengsheng He, Qihong Zhao

**Affiliations:** ^1^Department of Pathology, Lujiang County People's Hospital, Hefei, Anhui, China; ^2^Department of Nutrition and Food Hygiene, School of Public Health, Anhui Medical University, Hefei, Anhui, China; ^3^School of Public Health, Wannan Medical College, Wuhu, Anhui, China; ^4^Technology Center of Hefei Customs, Anhui Province Key Laboratory of Analysis and Detection for Food Safety, Hefei, Anhui, China; ^5^Department of Nutrition and Food Hygiene, School of Public Health, Fudan University, Shanghai, China

**Keywords:** BKMR, WQS, GC, combined effect, case-control study

## Abstract

**Background:**

Limited epidemiological evidence suggests that exposure to trace elements adversely impacts the development of gastric precancerous lesions (GPL) and gastric cancer (GC). This study aimed to estimate the association of individual urinary exposure to multiple elements with GPL and GC.

**Methods:**

A case-control investigation was conducted in Anhui Province from March 2021 to December 2022. A total of 528 subjects (randomly sampled from 1,020 patients with GPL, 200 patients with GC, and 762 normal controls) were included in our study. Urinary levels of iron (Fe), copper (Cu), zinc (Zn), nickel (Ni), strontium (Sr), and Cesium (Cs) were measured using inductively coupled plasma mass spectrometry (ICP-MS). Four different statistical approaches were employed to explore the risk of GPL and GC with mixed exposure, including multivariate logistic regression, weighted quantile regression (WQS), quantile g-computation (Qgcomp), and the Bayesian kernel machine regression (BKMR) model.

**Results:**

The WQS model indicated that urinary exposure to a mixture of elements is positively correlated with both GPL and GC, with ORs for the mixture exposure of 1.34 (95% CI: 1.34-1.61) for GPL and 1.38 (95% CI: 1.27-1.50) for GC. The Qgcomp and BKMR models also demonstrated a statistically significant positive correlation between the mixture and both GPL and GC.

**Conclusion:**

Considering the limitations of case-control studies, future prospective studies are warranted to elucidate the combined effects and mechanisms of trace elements exposure on human health.

## 1 Introduction

Recent surveys have found that gastric cancer (GC) accounts for 7.7% of all deaths and 22.2 million disability-adjusted life years (DALYs), highlighting its growing significance as a global health concern (1). Gastric precancerous lesions (GPLs) represent the pathological transformation process from normal gastric mucosa to GC, involving a multistep process that includes chronic atrophic gastritis, intestinal metaplasia, and atypical hyperplasia (2). The occurrence of chronic atrophic gastritis and intestinal metaplasia exhibits substantial variation among populations, being more prevalent in East Asia, Eastern Europe, and South America regions, where the incidences of GC are higher (3–6). Environmental factors have been suggested to play a substantial role in the development and progression of both GC and GPL (7). Increasing epidemiological evidence indicates that environmental pollution caused by exposure to metals can contribute to the frequency of GC (8).

There are two major potential mechanisms by which commonly recognized heavy metals, i.e., lead (Pb), cadmium (Cd), mercury (Hg), chromium (Cr), and arsenic (As), induce gastric cancer (9). First, heavy metals (e.g., cadmium: Cd^2+^) can displace calcium (Ca^2+^) and affect the functions of E-calmodulin, thereby disrupting cellular connectivity. This disruption can decrease mucus thickness and content, reduce basal acid output, and ultimately increase the lipid peroxidation process, rendering the gastric mucosa more vulnerable. This may represent the key step in initiating GC and promoting cancer development (9). Second, heavy metals are important sources of exogenous reactive oxygen species (ROS). For example, cadmium displaces Fe and Cu in various cytoplasmic and membrane proteins, thereby increasing the amount of unbound, free, or chelated Fe and Cu ions, which are then involved in oxidative stress via the Fenton reaction (10). When the level of ROS accumulation exceeds the capacity for antioxidant protection, gene regulation is altered, resulting in DNA damage, DNA repair failure, telomere dysfunction, and mitotic abnormalities. Additionally, carcinogenic metals (e.g., As and Cr) induce post-translational histone modifications and regulate histone-modifying enzymes, including the Fe- and 2-oxoglutarate-dependent dioxygenase family of enzymes, the DNA repair enzymes ABH3 and ABH2, and the histone methyltransferases, potentially influencing the epigenome (11). These processes collectively impact cell growth, differentiation, and apoptosis, leading to inflammation and damage to the endothelial and epithelial barriers, ultimately inducing carcinogenesis.

Apart from the recognized carcinogenic heavy metals, there is a growing research of interest in trace elements such as Fe, Cu, Zn, Ni, Sr, and Cs. These elements are indispensable components of many enzymes and proteins and participate in many physiological functions, including the synthesis of biological macromolecules, oxygen transport in the body, antioxidant function, and maintenance of immune function (12, 13). Excessive intake of certain trace elements is known to potentially disrupt gastrointestinal function, contributing to disorders such as GC (14). However, studies have been inconsistent regarding the relationship between the aforementioned trace elements and GC (15). For example, data from an epidemiological study in Iran suggested that higher Zn levels are associated with a reduced risk of GC (16). Conversely, a meta-analysis reported no statistically relevant association between Zn levels and the risk of GC (17). Moreover, Lin et al. reported significantly increased concentrations of Cu in GC cases (18). Similarly, a case–control study demonstrated an inverse relationship between Fe levels and GC (19), while a randomized controlled trial showed inconclusive results (20). In summary, although Fe may be biologically relevant to gastric cancer, the results are heterogeneous and insufficient to draw definitive conclusions (21). A recent report discovered that the concentration of Cs in urine may be negatively correlated with interleukin (IL)-6, which has been proven to activate key signaling pathways in the progression of gastritis and GC (22, 23). Animal studies suggest that cesium chloride at doses ranging from 0.8 to 1.2 g/kg may inhibit tumor proliferation in nude mice (24).

Therefore, it is necessary to establish a scientific method to study the effects of exposure to various trace elements on GC and GPL. Previous epidemiological evidence has primarily focused on the association between single trace elements and GC, largely ignoring the potential impact of trace elements exposure on GPL. However, in nature, humans can be simultaneously exposed to a wide range of elements, and the impact of a single element may underestimate the true impact of possible interactions with other metals (25). Therefore, our research was conducted to explore the relationship between the mixed exposure of trace elements and the occurrence of GC and GPL in the locals of Anhui province, which has exhibited very high prevalence and mortality rates of GC and GPL (26).

## 2 Methods

### 2.1 General demographic characteristics

All newly diagnosed GC and GPL cases from Lujiang County People's Hospital were recruited from March 2021 to December 2022. All cases who were aged 18 years or older were eligible for our study. GC and GPL were defined according to the UICC classification. Newly diagnosed GC and GPL cases were collected from the Department of Gastroenterology, and comprehensive pathological reports were obtained for each participant. Patients who had received gastrointestinal medications, had mental illnesses, were diagnosed with other tumors, and missed the data for the baseline survey, urinary metals, or creatinine were excluded from our analysis.

Questionnaires and urine samples were randomly recruited from a total of 1,982 participants (1,020 patients with GPL, 200 patients with GC, and 762 normal controls). GPL and GC cases and healthy controls were matched by gender and age (±3 years) using propensity score matching (PSM). Controls were selected from the hospital's health management center and were admitted simultaneously with the cases. Comprehensive medical examinations confirmed that controls had no stomach problems, gastrointestinal diseases, mental illnesses, or tumors, and had not received gastrointestinal medications. All participants signed an informed consent document, and the study was approved by the Ethics Committee of Anhui Medical University (No. 20190292). In total, 528 people were enrolled: 175 with GPL, 175 controls, and 178 with GC. The flowchart of recruiting study subjects is illustrated in Supplementary Figure S1.

### 2.2 Sampling of urine and measurement of urinary metals

The morning urine samples from each subject were collected in a 40 ml polypropylene (PP) tube, centrifuged at 1,350 × *g* for 8 min, and the supernatant was transferred to a new 5 ml PP tube and stored in the laboratory refrigerator for further testing.

Inductively coupled plasma mass spectrometry (ICP-MS; Perkin Elmer NexION 350X) was used to test for six trace elements in urine: Fe, Cu, Zn, Ni, Sr, and Cs. These elements were selected for their potential relevance in the progression of GC based on recent reports. The 29-element stock solution (10 μg/ml; PE#: N9300233) and mix internal standards (10 μg/ml) (PE#: N9303832) were purchased from Perkin Elmer Life and Analytical Sciences. Triton^®^ X-100 was sourced from Sigma-Aldrich Co. Up-s grade (ultra-pure) nitric acid (HNO_3_) was obtained from Suzhou Crystal Clear Chemical Corporation (Suzhou, China).

Before testing, urine samples were thawed at 4°C, placed into a 50 ml sterile PP tube, and diluted 10-fold with a mixture containing 1% (v/v) HNO_3_ and 0.05% Triton X-100. Multi-element working standards were prepared for each run by serially diluting the standard stock solution with a diluent solution containing 1% (v/v) nitric acid and 0.05% (v/v) Triton. The standard curve ranged from 0 to 50 μg/L for the six biomonitoring methods. Yttrium, a gray-black metal with the chemical symbol Y, was used as the internal standard. A blank sample was tested as QC for every 20 samples. The detailed data regarding the limit of detection (LOD) can be found in Supplementary Table S2. The precision and recovery rate of the elements met experimental requirements, with a recovery rate of 81.28%−116.79% (Supplementary Table S3).

Urinary creatinine content was used to normalize the concentration of elements in urine. The sarcosine oxidase method was used to detect urinary creatinine concentration with an automated biochemical analyzer (BS200, Mindray Bio-medical Electronics Co. LTD, Shenzhen, China) and strictly followed the manufacturer's instructions. The creatinine assay kit was purchased from Nanjing Jiancheng Bioengineering Institute.

### 2.3 Covariates

Covariates included age, gender (male/female), body mass index (BMI) (underweight, normal weight, overweight, and obesity), education (primary school, junior school, and high school or above), occupation (farmer or non-farmer), income (CNY; < 10,000, 10,000–60,000, and ≥60,000), family history of gastric cancer (Yes or No), tobacco smoking (Yes or No), alcohol drinking (Yes or No), and *Helicobacter pylori* infection (C_14_ breath test and the colloidal gold labeling method; Aibo Biomedical Co., Ltd, Hangzhou, China). Height and weight were measured, and BMI was categorized as underweight (BMI ≤ 18.5), normal weight (BMI 18.5–24.5), overweight (BMI 24.5–28.0), and obesity (BMI ≥28.0). Smoking was defined as smoking at least one cigarette a day for over 6 months. Drinking was defined as consuming alcohol at least three times a week for over 6 months.

### 2.4 Statistical analysis

A descriptive analysis of the demographic characteristics and trace elements' concentration of all eligible subjects was performed, and the distribution of elements was described using the median and quartile range. The *T*-test was used for comparisons for normally distributed measurements, while non-parametric analyses were used for non-normally distributed data. Categorical information was calculated using the Chi-square test. Spearman's rank correlation test was performed to assess the potential correlation between element concentrations in urine and GC and GPL.

#### 2.4.1 Multivariate logistic regression

The association between single-element exposure and GPL and GC was analyzed using a multivariate logistic regression model. Model 1 included only single creatinine-adjusted elements, while model 2 was adjusted for gender, age, BMI, education level, income, occupation, family history of GC, tobacco smoking, alcohol drinking, and *H. pylori* infection at baseline. The lowest quantiles were used as reference. Possible non-linear relationships between urinary metals and GPL and GC were explored with restricted cubic spline (RCS).

#### 2.4.2 WQS regression model

Weighted quantile sum (WQS) regression is an innovative model designed for multiple regression in high-dimensional datasets (27), with indices assessing the impact of all exposure variables on outcomes. In addition, the weights of each exposure variable can be used to assess its relative importance (28). WQS regression assumes that all exposure variables are associated with outcomes in the same direction, either positive or negative. This study applied four sets of WQS regression models to assess the link between trace elements mixtures and GC and GPL in different directions. Additionally, positive and negative constraints were applied for weight assessment.

#### 2.4.3 Quantile g-computation

Considering the non-linear effects and exposure correlation of multiple trace elements exposure on GC and GPL, Quantile g-computation (Q-gcomp) was used to analyze the effects of elements mixture. Q-gcomp simultaneously estimates the impact of increasing each exposure by one quantile, combining the simplicity of reasoning with high computational efficiency. It also allowed “weight” to move in either a positive or negative direction (29).

#### 2.4.4 Bayesian kernel machine regression

Bayesian kernel machine regression (BKMR) was used to estimate the combined effects of trace elements mixtures on GC and GPL, accounting for possible non-linear interactions between elements, based on the formula (30): Yi = h (Fei, Cui, Zni, Csi, Sri, Nii) + xi'β + εi, where Yi refers to the risk of GC and GPL, h () represents the exposure–response function to be evaluated, Xi indicates the influence of a series of covariates, and εi represents residuals.

The BMKR model can also calculate posterior inclusion probability (PIP), providing a reference for determining the importance of metals' exposure to the results (31). The interactions between the trace elements were further analyzed by dividing the individual elements into high and low groups based on median trace element content, thus creating variables. Additive and multiplicative interactions were also analyzed.

R software was used for all analyses, with two-tailed *p*-values and a statistical significance threshold set at 0.05. The R packages “gWQS,” “qgcomp,” and “bkmr” were used to construct the mixed exposure models.

## 3 Results

### 3.1 General population characteristics

Our study included a total of 528 participants, comprising 178 gastric cancer (GC) patients, 175 gastric precancerous lesions (GPL) patients, and 175 controls. Compared to the controls, both GC and GPL patients had lower income and education levels and were more likely to be farmers. Both GC (90 of 178, 50.6%) and GPL (99 of 175, 56.6%) had a higher prevalence of *H. pylori* infection than controls (66 of 175, 37.7%). Furthermore, a higher proportion of GC (20.2%) and GPL (23.2%) patients had a family history of GC compared to controls (4%; Table 1).

**Table 1:** Baseline characteristics of subjects in GPL and GC and control groups.

**Characteristics**	**Categories**	**Control (%) (*n* = 175)**	**GPL (%) (*n* = 175)**	**GC (%) (*n* = 178)**	***p*-value^a^**	***p*-value^b^**
Sex	Male	107 (61.1)	110 (62.9)	111 (62.4)	0.900	0.826
	Female	68 (38.9)	65 (37.1)	67 (37.6)		
Age (years)		56.37 ± 8.50	56.77 ± 9.50	62.80 ± 10.42	**< 0.001**	0.683
BMI (kg/m^2^)	< 18.5	119 (68.0)	14 (8.0)	18 (10.1)	**< 0.001**	**< 0.001**
	18.5–24.5	48 (27.4)	93 (53.1)	125 (70.2)		
	24.5–28.0	8 (4.6)	43 (24.6)	27 (15.2)		
	> 28.0	0 (0.0)	25 (14.3)	8 (4.5)		
Education	Primary School	54 (30.9)	106 (60.6)	104 (58.4)	**< 0.001**	**0.010**
	Junior School	27 (15.4)	38 (21.7)	42 (23.6)		
	High school and above	94 (53.7)	31 (17.7)	32 (18.0)		
Occupation	Farmer	61 (34.9)	88 (50.3)	88 (49.4)	**0.008**	**0.003**
	Non-Farmer	114 (65.1)	87 (49.7)	90 (50.6)		
Annual household income	< 10,000	73 (41.7)	57 (32.6)	95 (53.4)	**< 0.001**	**< 0.001**
	10,000–60,000	41 (23.4)	105 (60.0)	70 (39.9)		
	>60,000	61 (34.9)	13 (7.4)	13 (7.3)		
Smoking	Yes	68 (38.9)	59 (33.7)	80 (44.9)	0.293	0.300
	No	107 (61.1)	116 (66.3)	98 (55.1)		
Drinking	Yes	63 (36.0)	68 (38.9)	62 (34.8)	0.906	0.590
	No	112 (64.0)	107 (61.1)	116 (65.2)		
Family history of gastric cancer	Yes	7 (4.0)	41 (23.4)	36 (20.2)	**< 0.001**	**< 0.001**
	No	168 (96.0)	134 (76.6)	142 (79.8)		
*H. pylori* infection	Positive	66 (37.7)	99 (56.6)	90 (50.6)	**0.020**	**< 0.001**
	Negative	109 (62.3)	76 (43.4)	88 (49.4)		

BMI body mass index, GPL gastric precancerous lesions, GC gastric cancer.

^a^p-value between the control and GC using the chi-square test.

^b^p-value between the control and GPL using the chi-square test.

Bold indicates statistical significance (*p* < 0.05).

**Figure 1:**
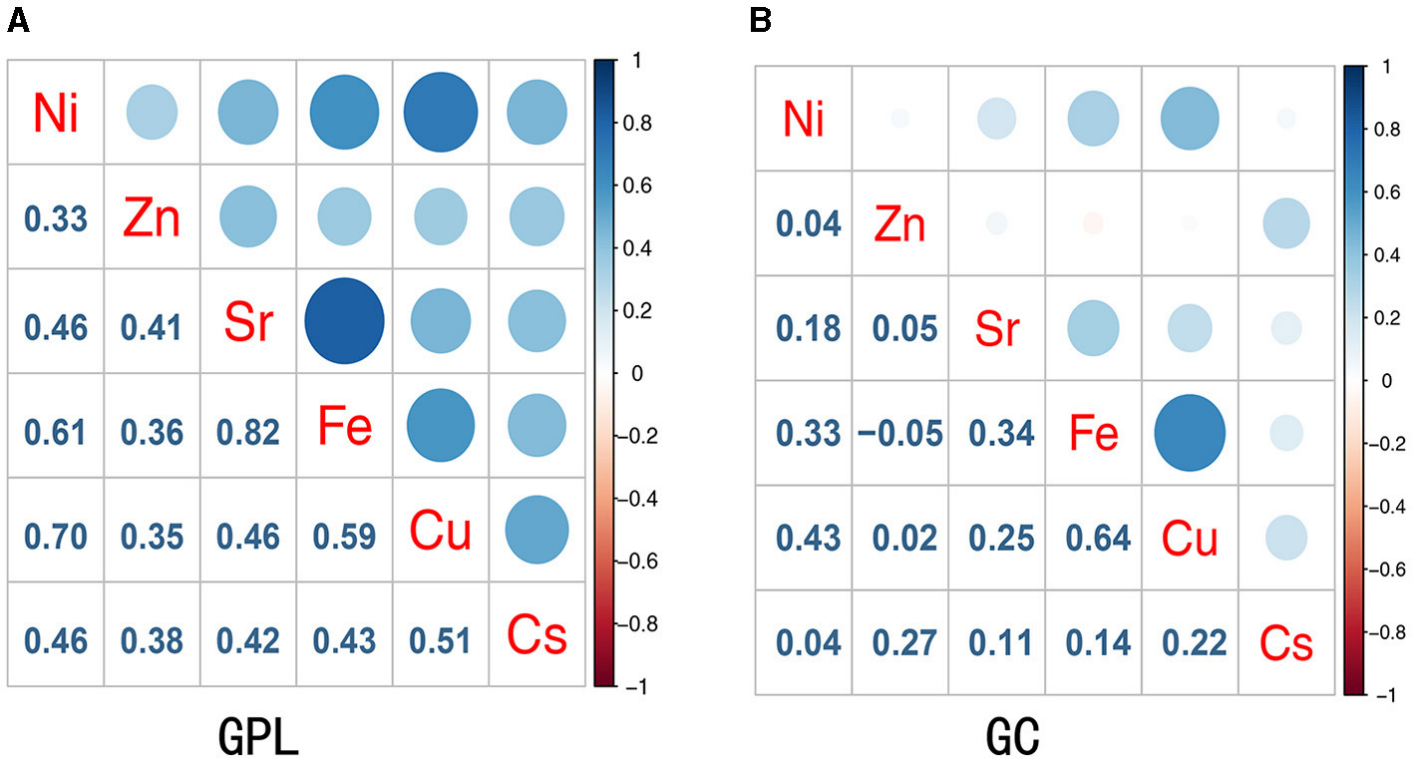
Spearman correlations between concentrations of urinary trace elements in the study participant. **(A)** Presented the correlation coefficients between metals in the GPL group and controls; **(B)** presented the correlation coefficients between metals in the GC group and controls.

The correlation between trace elements was examined using Spearman's rank correlation coefficient (Figure 1). Figure 1A presents the correlation coefficients between trace elements in the GPL group and controls. A significant positive correlation was observed ranging from 0.33 to 0.82 (*p* < 0.05), with the highest correlation between Fe and Sr (*r* = 0.82). The strongest association in the GC and control group was found between Fe and Cu (*r* = 0.64; Figure 1B). Therefore, a multi-contamination approach is required to investigate the effects of metallic mixtures on GPL and GC. The urinary metallic concentrations of the populations are detailed in Supplementary Table S1. Median urine concentrations of Zn and Ni in GPL (687.981 and 20.463 μg/g, respectively) and GC (633.665 and16.003 μg/g, respectively) groups were obviously increased compared with normal controls (455.448 and 8.592 μg/g, respectively; all *p* < 0.05), the distributions of Sr was similar between controls (159.471 μg/g) and all GC cases (201.689 μg/g; *p* < 0.05); however, median urine concentrations of Fe were all lower in GPL (117.993 μg/g) and GC (118.479 μg/g) groups than in controls (165.965 μg/g; all *p* < 0.05).

### 3.2 Single-element models

We analyzed the associations between urinary metal levels and GPL and GC using a multivariate regression model (Table 2). In the unadjusted single-element model (Model 1), log-transformed Sr, Zn, and Ni levels were significantly positively correlated with both GPL and GC. Conversely, an inverse association was found between log-transformed Fe and GC and log-transformed Cs and GPL. In the adjusted single-elements analysis (Model 2), statistically significant positive relationships persisted between Sr (OR for 4th quartile: 3.04, 95% CI: 1.60–5.78), Zn (OR for 4th quartile: 7.49, 95% CI: 3.46–16.20), and Ni (OR for 4th quartile: 2.65, 95% CI: 1.36–5.17) and both GPL and GC. The trends for log-transformed Sr, Zn, and Ni remained significantly positive in relation to GC risk. Similar results were found in Model 2, indicating a significantly negative correlation between urinary Fe and GC (OR for 4th quartile: 0.31, 95% CI: 0.17–0.58). However, urinary Cs levels were significantly related only to GPL cases (OR for 3rd quartile: 0.45, 95% CI: 0.05–0.70). RCS results indicated statistically non-linear associations for Zn, Cu, and Sr only in the GC group (with *p*-values for non-linearity of 0.038, 0.026, and 0.003, respectively; Supplementary Figure S2).

**Table 2:** Odds ratios [95% confidence interval (CI)] for the associations of GPL and GC with ln-transformed concentrations of urinary metals.

**Elements**	**GPL**		**GC**	
	**Model 1**	**Model 2**	**Model 1**	**Model 2**
**Zn**				
Q1	Ref.	Ref.	Ref.	Ref.
Q2	2.50 (1.32–4.73)	2.44 (1.27–4.70)	2.89 (1.56–5.45)	2.57 (1.56–5.45)
Q3	3.54 (1.82–6.88)	3.49 (1.73–7.03)	3.32 (1.79–6.28)	3.32 (1.79–6.28)
Q4	6.74 (3.25–13.99)	7.49 (3.46–16.20)	4.28 (2.30–8.16)	4.28 (2.30–8.16)
*p*-value	**< 0.001**	**< 0.001**	**< 0.001**	**< 0.001**
**Sr**				
Q1	Ref.	Ref.	Ref.	Ref.
Q2	1.23 (0.65–2.33)	1.15 (0.59–2.22)	0.78 (0.42–1.43)	0.86 (0.47–1.56)
Q3	2.11 (1.11–4.02)	1.98 (1.02–3.82)	1.12 (0.62–2.05)	1.08 (0.59–1.96)
Q4	3.06 (1.65–5.67)	3.04 (1.60–5.78)	2.41 (1.31–4.50)	2.35 (1.28–4.37)
*p*-value	**< 0.001**	**< 0.001**	**0.005**	**0.005**
**Ni**				
Q1	Ref.	Ref.	Ref.	Ref.
Q2	0.79 (0.41–1.51)	0.76 (0.39–1.49)	3.47 (1.82–6.81)	3.40 (1.78–6.70)
Q3	0.90 (0.50–1.63)	0.91 (0.50–1.68)	6.05 (3.16–12.02)	5.95 (3.09–11.84)
Q4	2.84 (1.35–5.44)	2.65 (1.36–5.17)	7.91 (4.09–15.90)	7.83 (4.04–15.74)
*p*-value	**0.001**	**< 0.001**	**< 0.001**	**< 0.001**
**Fe**				
Q1	Ref.	Ref.	Ref.	Ref.
Q2	0.95 (0.53–1.69)	0.84 (0.45–1.54)	0.75 (0.41–1.38)	0.75 (0.40–1.38)
Q3	0.85 (0.47–1.52)	0.71 (0.38–1.33)	0.40 (0.21–1.72)	0.39 (0.21–0.72)
Q4	1.20 (0.67–2.14)	1.28 (0.69–2.35)	0.32 (0.17–0.59)	0.31 (0.17–0.58)
*p*-value	0.70	0.72	**< 0.001**	**< 0.001**
**Cu**				
Q1	Ref.	Ref.	Ref.	Ref.
Q2	0.86 (0.48–1.52)	0.85 (0.47–1.55)	1.70 (0.67–4.41)	1.43 (0.78–2.63)
Q3	1.06 (0.59–1.92)	1.06 (0.58–1.96)	1.93 (0.80–4.77)	1.23 (0.68–2.24)
Q4	1.34 (0.74–2.42)	1.17 (0.63–2.17)	0.90 (0.35–2.31)	0.74 (0.40–1.35)
*p*-value	0.50	0.54	0.11	0.11
**Cs**				
Q1	Ref.	Ref.	Ref.	Ref.
Q2	0.58 (0.32–1.06)	0.54 (0.29–1.02)	0.50 (0.27–0.91)	0.46 (0.25–0.84)
Q3	**0.49 (0.27–0.89)**	**0.45 (0.24–0.85)**	0.73 (0.40–1.31)	0.66 (0.36–1.21)
Q4	1.29 (0.68–2.45)	1.22 (0.62–2.39)	0.68 (0.37–1.22)	0.64 (0.35–1.16)
*p*-value	**0.004**	**0.004**	0.16	0.16

Model 1 was the crude hazard ratios (95% CI).

Model 2 was adjusted for sex, age, body mass index, education, income level, occupation, smoking status, drinking, family history of gastric cancer, and H. pylori infection.

Q, quartile; Zn, Zinc; Sr, strontium; Ni, Nickel; Fe, Iron; Cu, Copper; Cs, cesium.

Bold indicates statistical significance (p < 0.05).

### 3.3 Combined effect of metal mixtures

**Figure 2:**
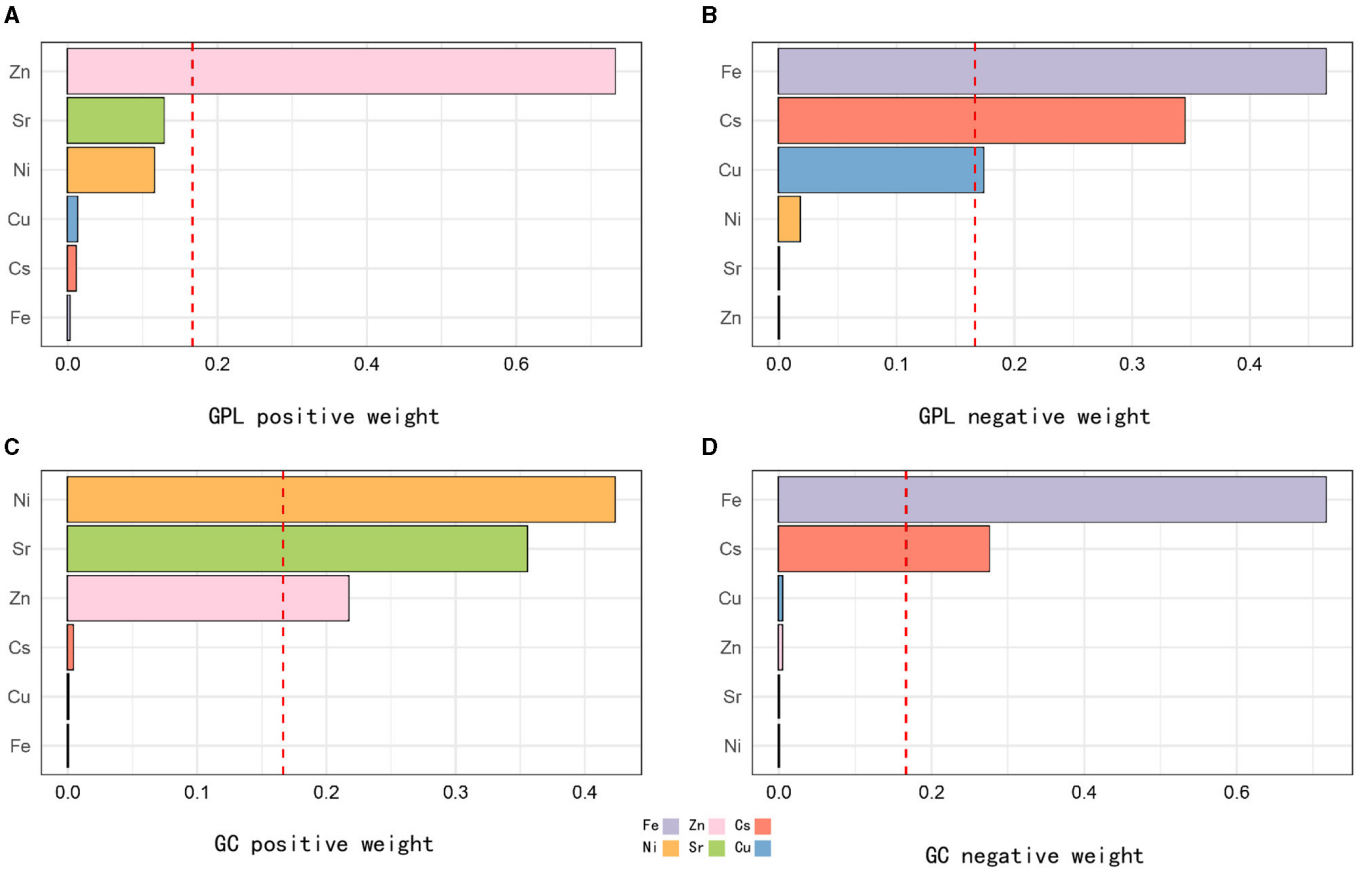
WQS model regression index weights for GPL and GC. The relative contribution of each trace element to the total effect of the mixture in urine with positive and negative associations with GPL **(A, B)** and GC **(C, D)**. The model was adjusted for sex, BMI, age, education, income level, occupation, family history of GC, lifestyle (such as smoking and drinking), and *H. pylori* infection. Q, quartile; Zn, Zinc; Sr, strontium; Ni, Nickel; Fe, Iron; Cu, Copper; Cs: cesium.

WQS regression models were used to examine the combined metal exposure in relation to GC and GPL. In the GPL and control group, the WQS regression index was positively related to GPL (OR: 1.34, 95% CI: 1.34–1.61; Table 3). Zn had the highest positive correlation coefficient (0.732), followed by Ni (0.344) and Sr (0.128). The negative correlation was mainly driven by Fe (0.464), followed by Cs (0.344) and Cu (0.174; Figures 2A, B). In the GC and control group, the WQS regression index also exhibited a positive correlation with GC (OR: 1.38, 95% CI: 1.27–1.50; Table 3). The weight of Ni was the highest when the WQS model was positive fitting (0.428), followed by Sr (0.348) and Zn (0.221). In contrast, the negative correlation was primarily driven by Fe (0.701), followed by Cs (0.290) and Cu (0.006; Figures 2C, D).

**Table 3:** ORs (95% CIs) for the risk of GPL and GC with log-transformed urinary trace elements concentrations based on WQS and Qgcomp models.

	**OR (95% CI)**	***p*-value**
**WQS**		
GPL	1.34 (1.34–1.61)	**< 0.001**
GC	1.38 (1.27–1.50)	**< 0.001**
**Qgcomp**		
GPL	1.31 (1.25–1.38)	**< 0.001**
GC	1.47 (1.33–1.62)	**< 0.001**

The model was adjusted for sex, BMI, age, education, income level, occupation, family history of GC, lifestyle (such as smoking and drinking), and H. pylori infection.

OR estimates represent mean differences in metals when the WQS and Qgcomp index increased by one quartile.

Bold indicates statistical significance (p < 0.05).

**Figure 3:**
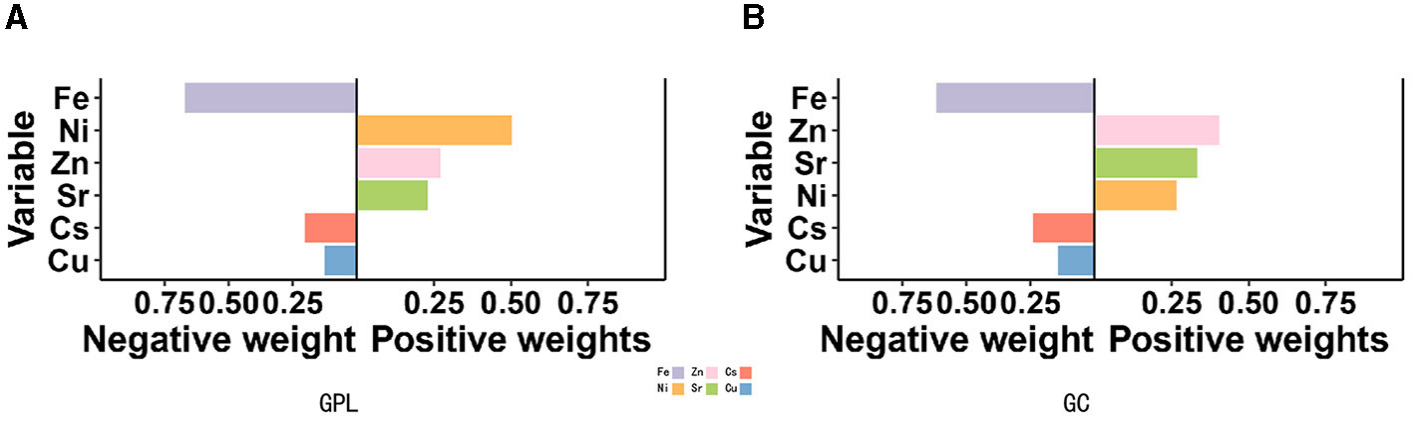
Association between Qgcomp index and GPL **(A)** and GC **(B)**. The model was adjusted for sex, BMI, age, education, income level, occupation, family history of GC, lifestyle (such as smoking and drinking), and *H. pylori* infection.

Quantile g-computation models were conducted to evaluate the combined exposure to elements in relation to GPL and GC. We found a strong positive relationship between multiple trace elements co-exposure and both GPL (OR: 1.31, 95% CI: 1.25–1.38) and GC (OR: 1.47, 95% CI: 1.33–1.62; Table 3). Zn, Ni, and Sr were significantly positively correlated with GPL and GC, while Fe, Cu, and Cs had negative effects (Figures 3A, B). The positive direction was driven by Zn (0.403) in GPL, followed by Sr (0.332) and Ni (0.264). In addition, Ni played a leading role in the GC development (X), followed by Zn (0.269) and Sr (0.229). The negative correlation was primarily driven by Fe in GPL (0.617) and GC (0.670), followed by Cs in GPL (0.245) and GC (0.203), and Cu in GPL (0.143) and GC (0.127; Supplementary Table S4).

**Figure 4:**
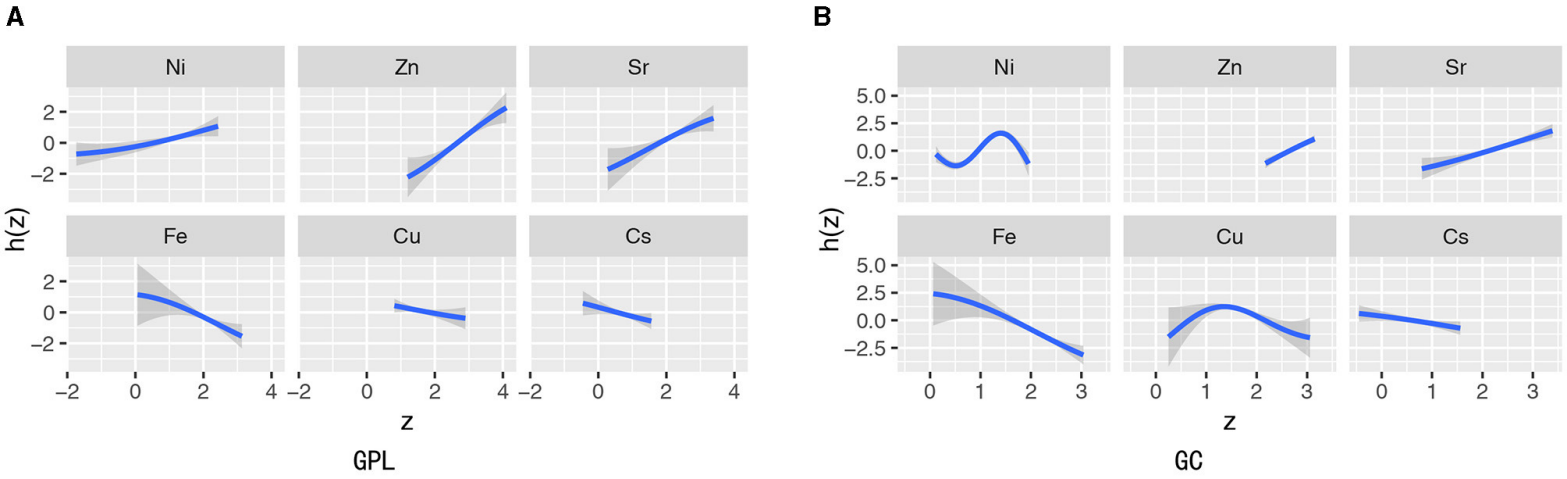
Univariate exposure–response functions and 95% confidence interval (gray part) for the association between urinary levels of single trace element exposure and odds of GPL **(A)** and GC **(B)** when concentrations of other elements were held at their median concentrations.

The BKMR method revealed the connection between each trace element and the risk of GPL and GC, illustrating the joint impact and interaction of elements. The number of iterations for the model is uniformly set to 10,000. Figure 4A shows that Ni, Sr, and Zn were positively associated with GPL, while Cs had an inverse trend. Figure 4B reveals that Sr, Zn, and Ni were positively correlated with GC, while Fe was strongly inversely correlated with GC, which was supported by the results in Supplementary Figure S3.

**Figure 5:**
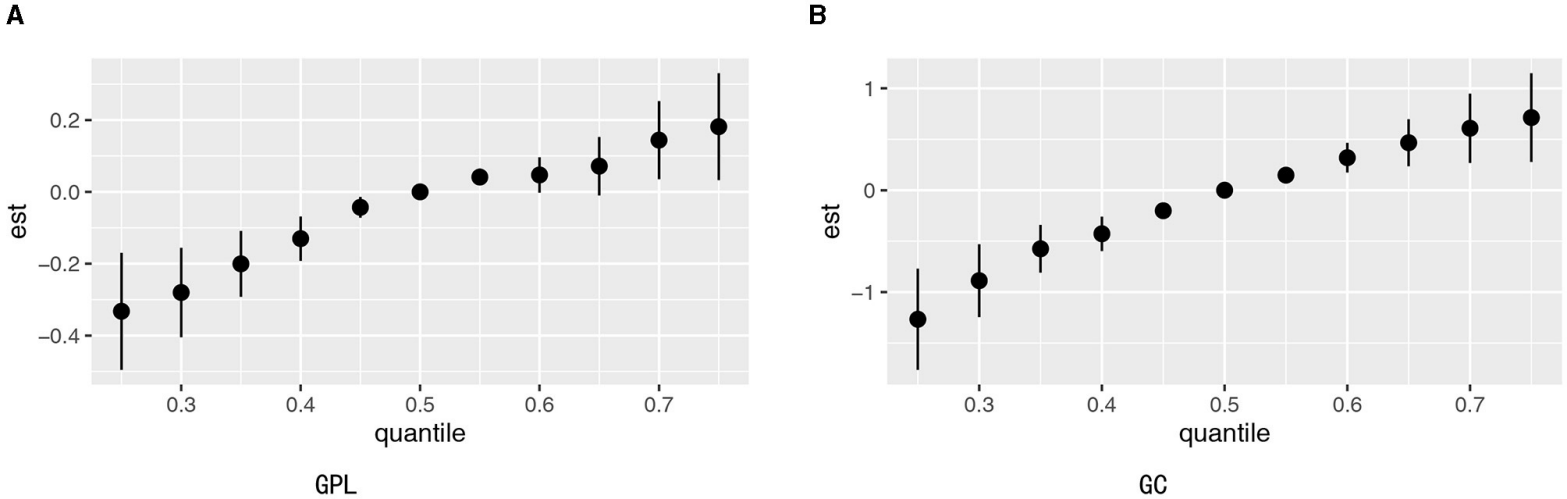
Estimated joint effects of trace elements mixtures on GPL **(A)** and GC **(B)** by BKMR. Adjusted variables included sex, BMI, age, education, income level, occupation, family history of GC, lifestyle (such as smoking and drinking), and *H. pylori* infection.

Figures 5A, B present the combined effects of urinary elements' mixture exposure on GPL and GC. When all urinary elements were higher than the 55th percentile, six trace elements had positive combined effects both in GPL and GC. The PIPs in Supplementary Tables S4, S5 indicated that Zn contributed the highest to GPL, while Fe, Sr, Zn, and Ni made a high contribution to GC. The bivariate exposure–response functions of the trace elements exposure associated with GPL and GC are shown in Supplementary Figures S4, S5. In the joint association analysis of Cu and Ni, a significant p-interaction value was found (*p*-interaction = 0.04; Supplementary Table S6). Individuals with the highest Cu and Ni exposure had a high likelihood for GC (3.74, 95% CI: 1.50–9.87), with strong multiplicative and additive relationships between the two elements and GC (ORint = 7.06, 95% CI: 1.13–49.81, AP = 0.43, 95% CI: −0.19 to 1.05, *S* = 2.42, 95% CI: 0.38–15.39).

## 4 Discussion

In our study, we employed four novel analysis approaches to explore the impact of individual and combined trace elements on GPL and GC. We found that elevated levels of Zn, Ni, and Sr were correlated with a higher risk of GPL and GC in a multivariate logistic model after adjusting for potential confounding factors, whereas Fe was observed to have an inverse effect on GC, and Cs was inversely correlated with GPL. Furthermore, the WQS and Qgcomp models suggested the positive impact of six trace elements on GPL and GC. The positive association between mixture elements and GPL was mainly driven by Zn, followed by Sr and Ni, while the negative effect was driven by Fe. For GC, Ni played a leading role in its development, followed by Sr and Zn, with the negative correlation mainly driven by Fe, followed by Cs and Cu. In addition, our data for the BKMR model found that the six analyzed metals' mixture was significantly associated with GPL and GC risk.

Even after adjusting for covariates, the concentration of Zn in urine still exhibited a positive relationship with both GPL and GC. The mechanisms of Zn exposure posing a risk to GPL and GC are still uncertain. A cross-sectional investigation declaimed that the median concentration of Zn in GC patients was significantly higher compared to healthy controls (0.79 μg/ml vs. 0.37 μg/ml, *p* = 0.01) (16). Nozadi et al. found that those with GC had higher concentrations of Zn exposure than normal control (882.15 ± 172.4 μg/kg vs. 702.11 ± 169.31 μg/kg) (32). Recent clinical evidence also suggested that serum Zn concentration in GC patients was higher than in controls (33). However, another case–control study suggested that patients with gastrointestinal disorders had lower serum zinc levels than healthy subjects (albeit nearly significant) (19). Furthermore, a previous meta-analysis of 19 reports demonstrated an insignificant correlation between Zn exposure and GC (17). Zn is the key mediator in superoxide dismutase; therefore, the imbalance of Zn in the human body promoting gastrointestinal tumors may be related to the decrease in antioxidant defense and immune response, leading to DNA synthesis damage (34). Additionally, Wu et al. (35) provided evidence that the disorder of Zn homeostasis in the organism may lead to excessive production of Zn in urine.

Sr exists in almost all environmental media, including air, drinking water, soil, and food (36, 37). Although Sr is an indispensable trace element, excessive levels may pose a potential risk to the human body (38, 39). The carcinogenic properties of excessive Sr exposure can be explained from two aspects: first, similar to calcium, Sr can strengthen ERK1/2 -MAPK signaling pathways, as confirmed in animal experiments (40, 41). Second, the upstream regulatory RAS is also activated by Sr (42). There are relatively few national and international epidemiological studies exploring the effects of Sr on gastrointestinal cancers. In our study, urinary Sr was positively correlated with GPL and GC. Consistent with the present study, Nakaji et al. suggested that a higher gastric cancer mortality rate was positively associated with a higher concentration of Sr exposure in drinking water after adjusting for age in Japan (43). In the same study, Sr exposure levels in drinking water were strongly related to the incidence of colorectal cancer in women (43). Meanwhile, in a cross-sectional study in Iran, Keshavarzi et al. (44) reported that Sr levels exceeded the recommended maximum concentration level in drinking water, suggesting that the prevalence of esophageal cancer may be related to Sr.

Interestingly, we also observed that urinary Ni levels were positively related to GPL and GC both in single or mixed trace element exposure. Evidence from existing epidemiological data has demonstrated that exposure to Ni has a carcinogenic effect on the human body (45). The pathways of Ni likely involve a series of epigenetic processes, involving DNA methylation, post-transcriptional modification of histones, and regulation of non-coding RNA (46). A cross-sectional study in Turkey observed significantly higher Ni exposure in fruits and vegetables, which may be associated with GC prevalence (47). Reddy et al. (48) provided evidence that patients with stomach disorders have higher concentrations of tissue Ni than healthy controls, which is similar to the results above. Additionally, Sivulka et al. (49) reported that workers who refined Ni had increased odds of nasal and lung cancer. Ni acts as an essential factor in the pathogenicity of *H. pylori*, which requires Ni to colonize the stomach and is related to GC (50). Our results confirmed that high concentrations of Ni exposure in GC cases indicated Ni as a powerful carcinogen.

Fe is also an essential trace element, and humans are mainly exposed to Fe through food in their daily life (51). Fe is mainly absorbed through the intestine and excreted from the body via urine (52). In the multivariate regression model, urine Fe concentration was significantly negatively correlated with GC in both Model 1 and Model 2. Contrary to our findings, a case–control study suggested that patients with GC had elevated Fe levels compared to normal control (53). However, the current finding is similar to Fonseca-Nunes et al. (54), a prospective cohort study in Finland suggested an inverse association between Fe exposure and GC in men, albeit not statistically significant in trend analysis. A well-designed nested case–control study with an 11-year follow-up of 456 gastric cases also found a lower likelihood of gastric cancer with increased Fe storage (54).

Additionally, our study also noticed an insignificant negative relationship between urinary Cu exposure and increased odds of GPL and GC. Similar to our results, Sohrabi et al. (16) reported an insignificant relationship between Cu levels and GC. In 2022, a Turkish study found no statistically significant association between serum Cu and GC (19). Moreover, Reddy et al. (48) discovered lower concentrations of Cu in the stomach cancer group compared to healthy individuals (21.2 ± 3.4 vs. 63.5 ± 9.4 μg/g). However, Lin et al. observed a positive significant correlation between Cu exposure and GC in China (18). Meanwhile, Kohzadi et al. (53) and his peers suggested a higher concentration of tissue Cu in the case group vs. controls. There is controversy over the association between Cu exposure and GC, which might relate to factors such as the type of biological sample, collection time, metal detection methods, and types.

This study is the first to investigate the effect of Cs exposure on GPL and GC from an epidemiological perspective. Cs is derived from many sources, such as air, soil, water, and organisms. Metallic Cs is traditionally used in battery, optical instrument, and semiconductor industries. A recent animal experiment suggested that Cs inhibits the proliferation and migration of fibroblasts (55). Cs generally exist in the human body and are primarily excreted through urine (85%) (56). Therefore, it would be a reliable indicator to evaluate trace element levels in urine (57). However, evidence from previous investigations on the link between Cs and GC is limited. Our study found a significant correlation between Cs and GPL. Qin et al. claimed that Cs may have antioxidant and anti-inflammatory effects, possibly related to its regulation of intracellular oxidative stress and inflammatory response, and urinary Cs can serve as a biomarker for certain types of cancer in the future (57). Some reasonable biological mechanisms have been explored, such as Cs, a potassium channel blocker, inhibiting cell proliferation and tumor growth by regulating the G0/G1 transition in the cell cycle (58). More prospective studies are needed in the future to confirm this connection between Cs and GC.

In order to solve the problem of collinearity between multiple trace elements, we applied various statistical methodologies to calculate the possible effects of metal co-exposure on GPL and GC to ensure the reliability and repeatability of the results. The BKMR model, as a powerful method, overcomes the shortcomings of multicollinearity and model section error limitations in our study. The WQS and Qgcomp models have been generally employed to evaluate the link between elements of co-exposure and disease outcomes, such as cardiovascular disease (59). In our research, Qgcomp models provided a flexible method to explain the reliable correlation between multi-element exposure and GPL and GC. These models can be used simultaneously to mutually validate the results, providing guidance for local residents and serving as a reference for preventing GPL and GC.

However, our study has several limitations that should be considered when interpreting our results. First, given the nature of the case-control design, we are limited in inferring a causal relationship between urinary trace elements and GPL and GC. Consequently, additional prospective reports are needed for a more detailed analysis. Second, we only collected a morning urine sample from each participant instead of the first-morning urine or 24-h urine, which may lead to some deviation in our assessment. Finally, urinary trace elements may not be the best biomarkers for certain elements exposure, for example, blood elements might be more suitable for evaluating Fe exposure.

## 5 Conclusion

Our results revealed that Ni, Zn, and Sr were significant risk contributors to GPL and GC. Conversely, Fe was found to have a protective effect on GC, and Cs was inversely correlated with GPL. The trace elements mixture exposure in urine might be positively correlated with GC and GPL, with Ni, Sr, and Zn playing major roles in the increased risk of GPL and GC.

## Data availability statement

The raw data supporting the conclusions of this article will be made available by the authors, without undue reservation.

## Ethics statement

All participants signed an informative consent document and the study had the approval of the Ethics Committee of Anhui Medical University (No. 20190292). The studies were conducted in accordance with the local legislation and institutional requirements. Written informed consent for participation in this study was provided by the participants' legal guardians/next of kin.

## Author contributions

SQ: Conceptualization, Investigation, Software, Supervision, Writing – original draft, Writing – review & editing. FX: Conceptualization, Investigation, Writing – original draft, Writing – review & editing. MW: Investigation, Methodology, Writing – review & editing. MZ: Investigation, Methodology, Writing – review & editing. SD: Investigation, Methodology, Writing – review & editing. GJ: Investigation, Methodology, Writing – review & editing. XZ: Data curation, Investigation, Methodology, Writing – original draft. WC: Investigation, Methodology, Supervision, Writing – original draft. LW: Investigation, Methodology, Writing – review & editing. YutZ: Writing – review & editing, Investigation, Methodology. WW: Data curation, Methodology, Writing – review & editing. PO: Writing – review & editing, Supervision, Methodology. TW: Data curation, Investigation, Writing – review & editing. XL: Investigation, Methodology, Writing – original draft. YuZ: Data curation, Software, Writing – original draft. YL: Investigation, Methodology, Writing – original draft. AH: Investigation, Supervision, Writing – original draft. WY: Project administration, Validation, Writing – original draft. GH: Project administration, Supervision, Writing – original draft. QZ: Formal analysis, Funding acquisition, Project administration, Validation, Writing – original draft.
