# Supplementary material for: Association analyses between urinary concentrations of multiple trace elements and gastric precancerous lesions and gastric cancer in Anhui province, eastern China
Source: Front Public Health. 2024 Aug 15;12:1423286. doi: 10.3389/fpubh.2024.1423286 (PMC11363071; doi:10.3389/fpubh.2024.1423286)
Supplement: Supplementary file 1 [file Data_Sheet_1.PDF]

Supplementary Materials

Contents

Fig S1 Flowchart of study subject inclusion

Fig S2 The restricted cubic spline (RCS) for the association between trace elements and GC

Fig S3 Single metal association between urinary trace elements and GC

Fig S4 Bivariate exposure response functions for trace elements mixture exposure associated with GPL

Fig S5 Bivariate exposure response functions for trace elements mixture exposure associated with GC

Table S1 Concentrations of six trace elements in urine

Table S2 Linear ranges, regression equations, LOD of the targeted analytes in the urine

Table S3 Precisions and recoveries of six elements in urine

Table S4 Summary results from different models in GPL

Table S5 Summary results from different models in GC

Table S6 Adjusted odds ratios (95% CI) for incident GC according to the combined categories of urinary Cu and Ni

Fig S1

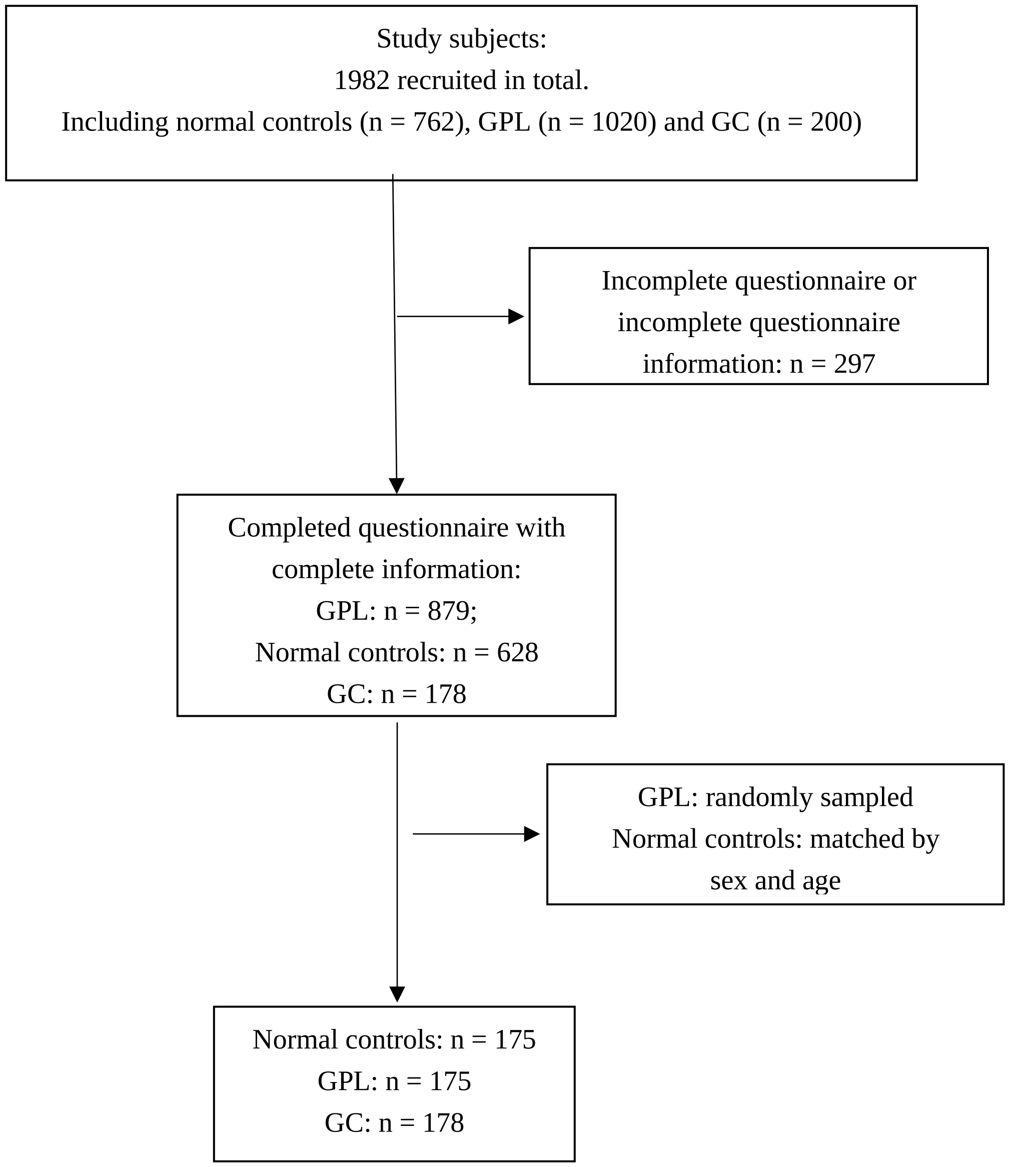

Fig S1 Flowchart of study subject inclusion

Fig S2

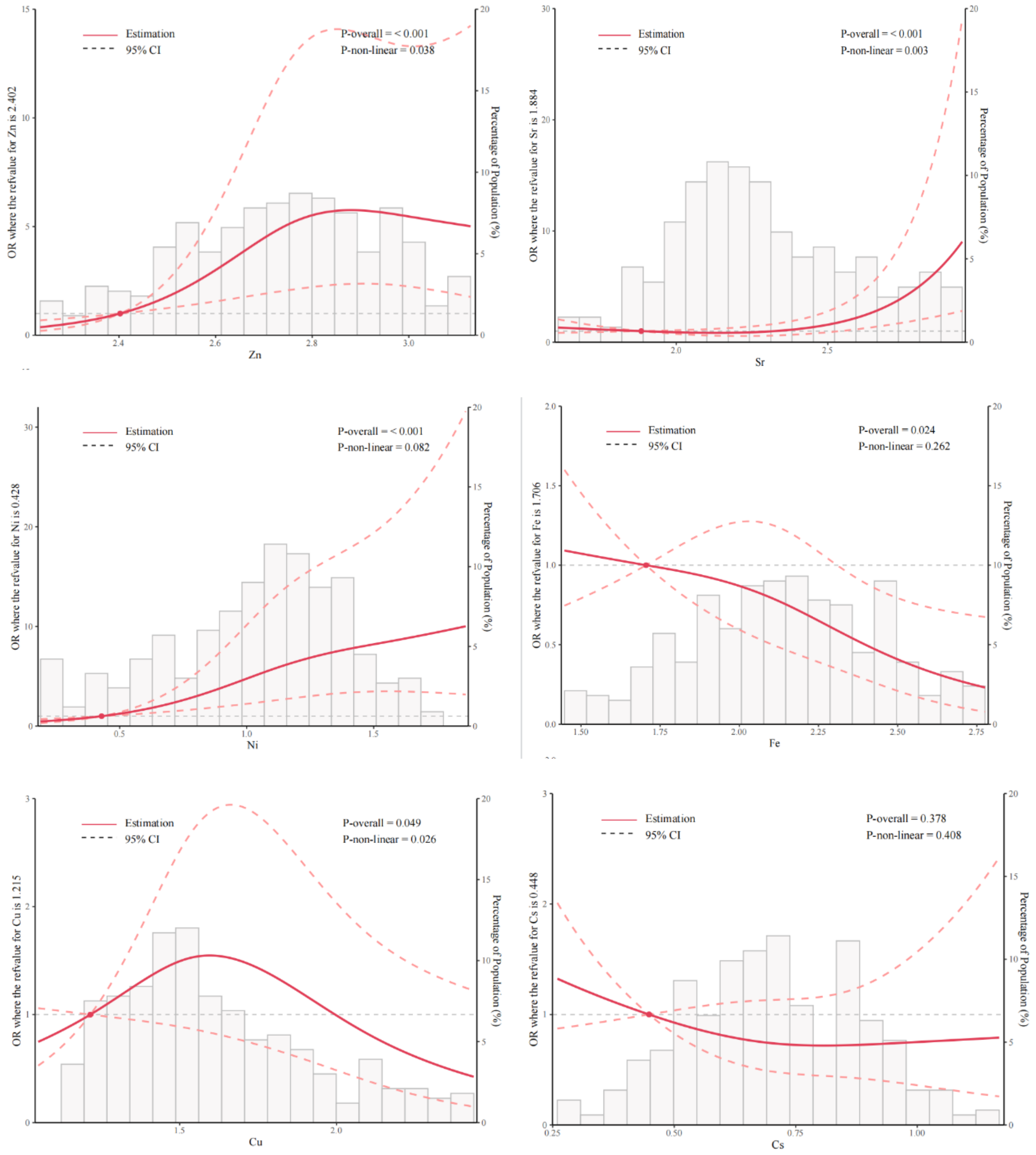

Fig S2 The restricted cubic spline (RCS) for the association between urinary trace elements and GC

The lines represent adjusted odds ratios [95% confidence interval (CI)] based on RCS for the log-transformed levels of six urinary **trace elements** in the multiple-elements model, with the reference value was set at the 10th percentile.

The model was adjusted for sex, BMI, age, education and income level, occupation, family history of GC and lifestyle (such as smoking and drink) and H. Pylori infection.

The bars represent histograms of urinary trace elements distribution among the participants.

Fig S3

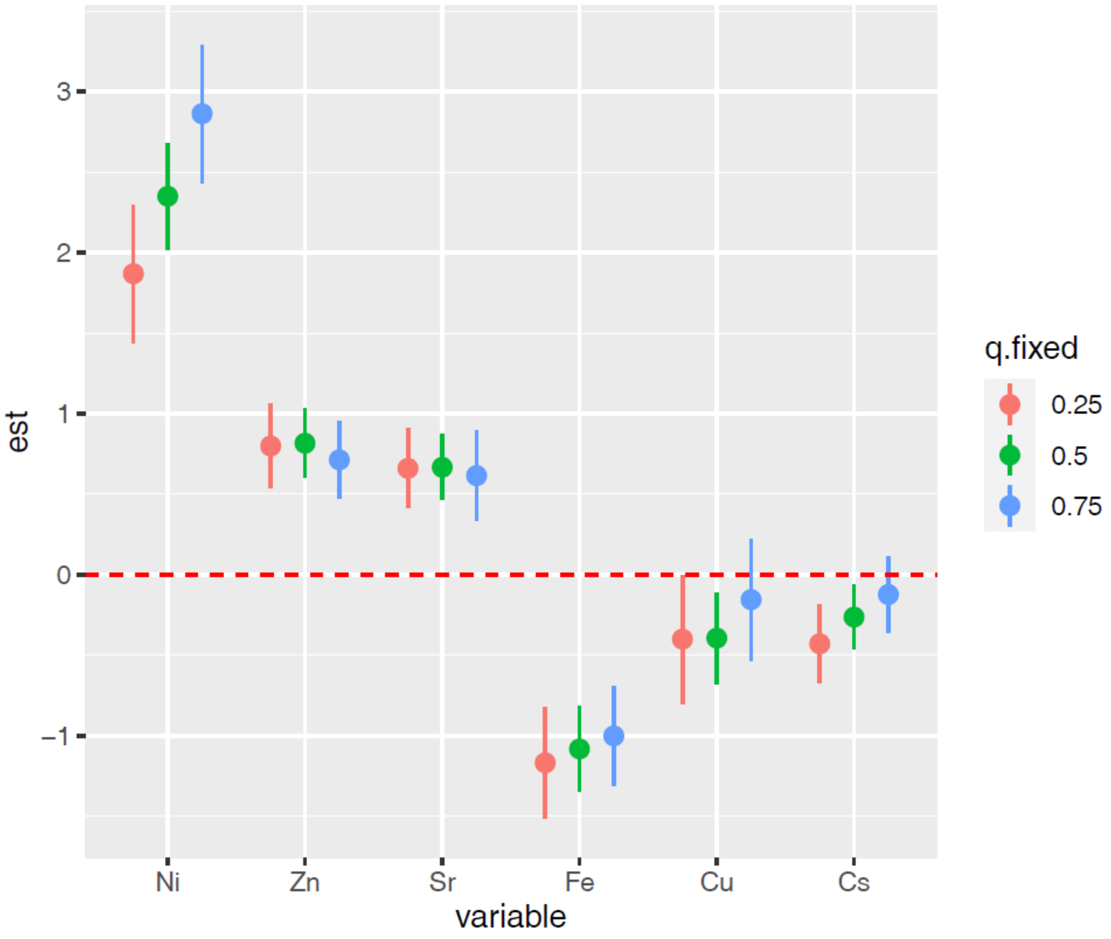

Fig S3 Single metal association between urinary trace elements and GC

This plot presented the effect of single metal by comparing the 75th of the metal concentrations with its 25th percentile, when concentrations of all the other metals were held at either the 25th (red line), 50th (green line), or 75th percentile (blue line).

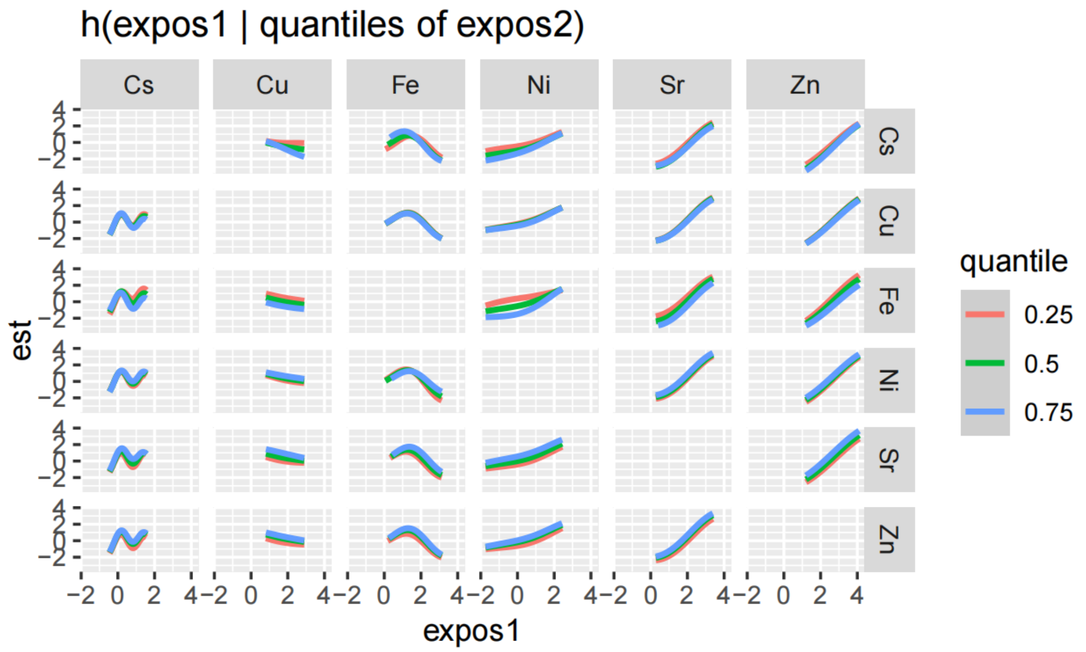

Fig S4 Bivariate exposure response functions for trace elements mixture exposure associated with GPL

For example: bivariate exposure Zn - Cs response function when copper is fixed at the 25th, 50th, or 75th percentile and other elements are fixed at intermediate values (Figure 1 on the right); Zn-Cu response function when Cu is fixed at the 25th, 50th, or 75th percentile and other elements are fixed at intermediate positions (second panel on the right); Zn - Fe response function when Fe is fixed at the 25th, 50th, or 75th percentile

Fig S5

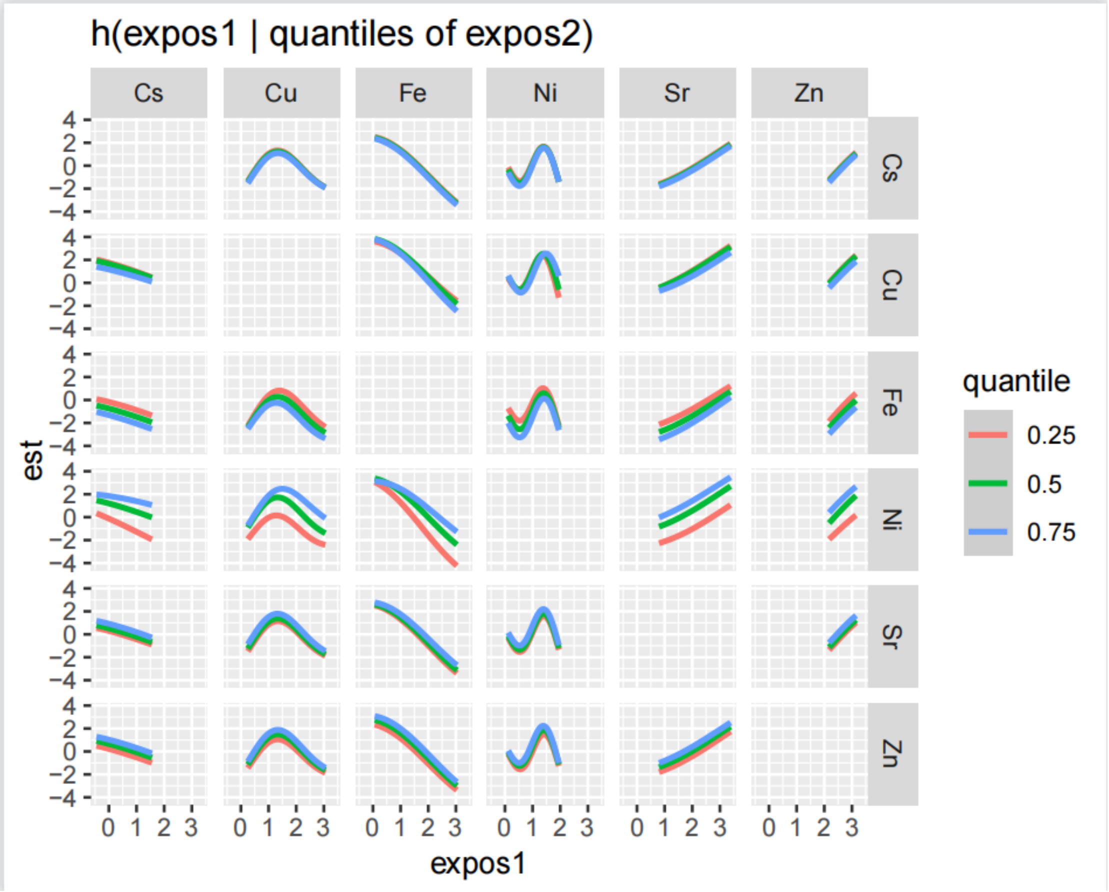

Fig S5 Bivariate exposure response functions for trace elements mixture exposure associated with GC

For example: bivariate exposure Zn - Cs response function when copper is fixed at the 25th, 50th, or 75th percentile and other elements are fixed at intermediate values (Figure 1 on the right); Zn-Cu response function when Cu is fixed at the 25th, 50th, or 75th percentile and other elements are fixed at intermediate positions (second panel on the right); Zn - Fe response function when Fe is fixed at the 25th, 50th, or 75th percentile.

Table S1 Concentrations of six trace elements in urine

Metals	Normal Controls (n = 175)	GPL (n = 175)	GC (n = 178)	*p* value[a]	*p* value[b]	*p* value[c]
Ni	8.592	20.463	16.003	<0.001	<0.001	<0.001
（median, IQR, μg/g)	（3.885-14.434）	（11.042-33.536）	（10.082-24.453）			
Zn	455.448	687.981	633.665	<0.001	<0.001	<0.001
（median, IQR, μg/g)	（308.784-692.695）	（506.003-985.788）	（468.124-886.588）			
Sr	159.471	166.652	201.689	0.001	0.880	0.001
（median, IQR, μg/g)	（110.747-255.166）	（98.149-289.535）	（118.864-454.935）			
Fe	165.965	117.993	118.479	<0.001	0.001	<0.001
（median, IQR, μg/g)	（105.708-276.728）	（68.817-217.384）	（69.215-195.941）			
Cu	36.680	37.062	33.5721	0.366	0.603	0.186
（median, IQR, μg/g)	（22.202-72.132）	（23.113-64.061）	（22.840-49.978）			
Cs	5.091	5.078	4.958	0.342	0.636	0.334
（median, IQR, μg/g)	（3.905-7.229）	（3.523-8.890）	（3.423-7.131）			

Abbreviations: IQR: Inter Quartile Range; Zn: Zinc; Sr: Strontium; Ni: Nickel; Fe: Iron; Cu: Copper; Cs: Cesium;

P value[a] was comparing the difference in concentration between the three groups with the Wilcoxon rank-sum test. *P* value[b] was comparing the difference in concentration between the normal controls and GPL group with the Wilcoxon rank-sum test. *P* value[c] was comparing the difference in concentration between the normal controls and GC group with the Wilcoxon rank-sum test.

Table S2

Linear ranges, regression equations, LOD of the targeted analytes in the urine

Analyte	Regression equation	Correlation coefficient	LOD (μg/L)
Fe	y=0.001x+0.006	0.999586	0.0594
Cu	y=0.053x+0.010	0.999733	0.0081
Zn	y=0.003x+0.004	0.999955	0.0308
Ni	y=0.022x+0.010	0.999941	0.0342
Sr	y=0.038x+0.011	0.999856	0.0042
Cs	y=0.065x-0.004	0.999914	0.0065

Table S3

Precisions and recoveries of six elements in urine

Element	Precision (RSD%)		Spiked (μg/L)	Recovery (%)
	Inter-day	Intra-day		
Fe	3.438	1.9040	1	84.19
			5	116.79
Cu	3.8574	2.2791	1	103.96
			5	102.61
Zn	4.0117	1.6472	1	88.01
			5	81.28
Ni	5.4158	2.3635	1	90.93
			5	97.80
Sr	1.6947	1.6285	1	94.65
			5	101.38
Cs	6.0163	5.6774	1	96.26
			5	84.31

Table S4

Summary results from different models in GPL

Variables	Multivariate logistic regression	WQS (Positive direction)	WQS (Negative direction)	Qcomp	BKMR (PIP)
Fe		0.002	0.464	0.617 (－)	0.950
Sr	＋	0.128	< 0.001	0.332 (＋)	0.979
Zn	＋	0.732	< 0.001	0.403 (＋)	1.000
Ni	＋	0.115	0.018	0.264 (＋)	0.949
Cs	－	0.011	0.344	0.245 (－)	0.891
Cu		0.012	0.174	0.143 (－)	0.826

Abbreviations: WQS: Weighted Quantile Sum; Qgcomp: Quantile g-computation; BKMR: Bayesian kernel machine regression. PIP: posterior inclusion probability;

Notes: *"+" means positive weight ; *"-" means negative weight.

The model was adjusted for sex, BMI, age, education and income level, occupation, family history of GC and lifestyle (such as smoking and drink) and H. Pylori infection.

Table S5

Summary results from different models in GC

Variables	Multivariate logistic regression	WQS (Positive direction)	WQS (Negative direction)	Qcomp	BKMR (PIP)
Fe	－	< 0.001	0.701	0.670 (－)	1.000
Sr	＋	0.348	< 0.001	0.229 (＋)	1.000
Zn	＋	0.221	0.004	0.269 (＋)	1.000
Ni	＋	0.428	< 0.001	0.502 (＋)	1.000
Cs		0.003	0.290	0.203 (－)	0.972
Cu		< 0.001	0.006	0.127 (－)	0.992

Abbreviations: WQS: Weighted Quantile Sum; Qgcomp: Quantile g-computation; BKMR: Bayesian kernel machine regression. PIP: posterior inclusion probability;

Notes: *"+" means positive weight ; *"-" means negative weight.

The model was adjusted for sex, BMI, age, education and income level, occupation, family history of GC and lifestyle (such as smoking and drink) and H. Pylori infection.

Table S6

Adjusted odds ratios (95% CI) for incident GC according to the combined categories of urinary Cu and Ni

	n (case/total)	odds ratios (95% *CI*)	OR_{int}	*P*-interaction
Low Cu+Low Ni	50/119	1.00	7.06(1.13,49.81)	0.04
Low Cu+High Ni	44/58	2.81 (1.36,5.98)		
High Cu+Low Ni	12/58	0.32(0.11,0.85)		
High Cu+High Ni	72/118	3.74(1.50,9.87)		
RERI: 1.60(-1.71,4.91), AP:0.43(-0.19,1.05), S:2.42(0.38,15.39)				

Abbreviations: Cu, Copper; Ni, Nickle.

The model was adjusted for sex, BMI, age, education and income level, occupation, family history of GC and lifestyle (such as smoking and drink) and H. Pylori infection.
